# Ecological assessment of Iran’s terrestrial biomes for wildlife conservation

**DOI:** 10.1038/s41598-023-45120-4

**Published:** 2023-10-18

**Authors:** Amir Ansari, Mansour Ghorbanpour, Ali Kazemi, Khalil Kariman

**Affiliations:** 1 Department of Environmental Sciences and Engineering, Faculty of Agriculture and Environment, Arak University, Arak, 38156-8-8349 Iran; 2 Department of Medicinal Plants, Faculty of Agriculture and Natural Resources, Arak University, Arak, 38156-8-8349 Iran; 3 UWA School of Agriculture and Environment, The University of Western Australia, Perth, WA 6009 Australia

**Keywords:** Ecology, Environmental sciences

## Abstract

Man-made activities pose the greatest threats to wildlife in Iran's terrestrial biomes, causing significant habitat damage and fragmentation in recent years. To fully understand these threats, the present study was conducted to identify and map the Iran’s terrestrial biomes using the IDRISI TerrSet 18.31 Software, the Terrestrial Biomes Ecosystem Service Modeler on the InVEST toolkit (TBESMI), and comprehensive data sources including maps of roads, protected areas, terrestrial biomes, and country-wide land cover maps of 2017. The results showed that the largest terrestrial biome in Iran is deserts and xeric shrublands (DXS), while flooded grasslands and savannas (FGS) is the smallest biome. Roads, along with urban and agricultural developments are among the biggest threats and most destructive stressors in Iran’s terrestrial biomes. The results also revealed that there was a growth in destruction of habitats located in the temperate broadleaf and mixed forest (TBMF), temperate coniferous forest (TCF), and FGS, alongside a decrease in the DXS biome. Furthermore, we detected an increase in habitat landscape quality in the DXS, FGS and montane grasslands and shrub lands (MGS), and a decrease in the temperate grasslands, savannas and shrublands (TGSS) and TBMF biomes. Finally, the cumulative risk of habitat degradation increased in the FGS, TCF, TGSS, and TBMF biomes, whereas it decreased in the DXS biome. The FGS biome with the highest consequence cumulative score, and the MGS biome with the highest cumulative risk exposure score were found to be at the highest risk from man-made activities. Stressors associated with agriculture and urbanization had the highest cumulative exposure scores in the MGS, while roads had the highest exposure scores in the TBMF and DXS biomes. Our study underscores the critical importance of conserving Iran's terrestrial biomes and wildlife, especially in high-risk biomes like FGS and MGS, given the substantial threats posed by human activities.

## Introduction

The 846 terrestrial ecoregions on Earth are classified into eight realms and fourteen biomes, six of which are forest biomes and eight are non-forest biomes^1^. In Iran, there are six important terrestrial biomes and six important terrestrial ecoregions^1^. Biodiversity in Iran is threatened, about 100 species of vertebrate fauna are vulnerable to or threatened with extinction. Man-made activities including urbanization, agriculture and industrial developments along with population growth, drought, desertification, and climate change have been the major contributors to the crisis. Biodiversity hotspots for endangered mammal species are located in the west, north, and center of Iran along the Alborz and Zagros mountains. Therefore, the habitats of endangered mammal species are restricted to relatively small areas of Iran (about 27% of the country). These regions are intensely fragmented and 57% of them are enunciated protected by the current conservation programs^2^.

We should prioritize terrestrial ecoregions located in the north, northwest, and west of the country in order to achieve the national goal of protecting 20% of Iran's land area. This is because these regions are important for biodiversity conservation, and increasing the coverage of protected areas in these regions would protect 70–100% of the distribution of most biodiversity groups, with the exception of birds and mammals, for which conservation cover would be lower^3^.

As sustainability of these habitats depends on the maintenance of scattered corridors to comfort the animals move among the habitat fragments, efforts of conservation should club on hotspots that are not officially protected under current laws of conservation^4^. The west and north of Iran are considered as the hotspots of Irano-Anatolian biodiversity, the so-called 20th region of global hotspot^4–6^. The mountain habitats in these areas are also defined by high levels of plant endemism, even higher than temperate European mountains such as the Alps^7,8^. As the snow line fell in Southwest Asia during the late Pleistocene glaciations, the wide Alborz and Zagros mountains emerged from the ice, leading to a downward migration of the Irano-Turanian high-altitude plant species^9^. The Alborz and Zagros mountain ranges are separated by arid lowlands, and this effective dispersal barrier intensified the escalation in the richness of the limited plant species in both ranges^10^.

Dispersal corridors are essential to facilitate the movement of animals among the habitats fragments, and that connectivity is essential to ensure the dispersal, conservation and survival of animal populations^11–13^. Connection adverts to the manufacture and force in which species, or social stagers diffuse, sources, habitats, interact or migrate among patches and social extents^14^.

Risk assessment of habitats should be implemented in the first stage of analyzing functional junctions at various scales, considering the patches with high human-related risks. The two implicates of connectivity assessment and habitat risk have never been used in to the framework of a biodiversity conservation. Since the 1950s, there has been an increase in the overall size of protected areas in Iran. However, a composition of many man-made factors threatens service capacity of the ecosystems^15,16^. The Service Modeler of Ecosystem is based on the toolkit of InVEST. The Rarity and Quality model evaluates the effects of man-made threats on the habitats rarity and quality. This model is generally used to determine sensitivity of habitats to landscape changes including land destruction and other stressors. Quality of habitat is determined through the proximity of land cover change and rarity of habitat by examining land degradation based on the historic status. Furthermore, this model enables a rapid evaluation of terrestrial habitat status, which can be used for in-depth investigation of the adaptability of species in an ecosystem^17^. The model also produces maps that highlight the changes of habitat due to the rarity and quality of the species over time. The Risk Assessment of Habitat model assesses the risks that depend on management strategies and man-made activities (including land use changes of agricultural, industrial, urban and rural areas). The model uses data from the given maps to assess the impact of man-made activities on an ecosystem, and the magnitude of risks caused by habitat changes depends on the extent to which the habitat is exposed to man-made activities and the consequences of that exposure. The database used for the current study comprised of the timing and severity of human activities, as well as the potential impact of different management methods, and utilized to produce maps showing the cumulative risks to specific habitats^18^. In the present study, we employed a novel model to thoroughly evaluate the ecological state of Iran's terrestrial biomes, aiming to provide valuable insights for future wildlife conservation initiatives.

## Material and methods

### Study area

**Figure 1:**
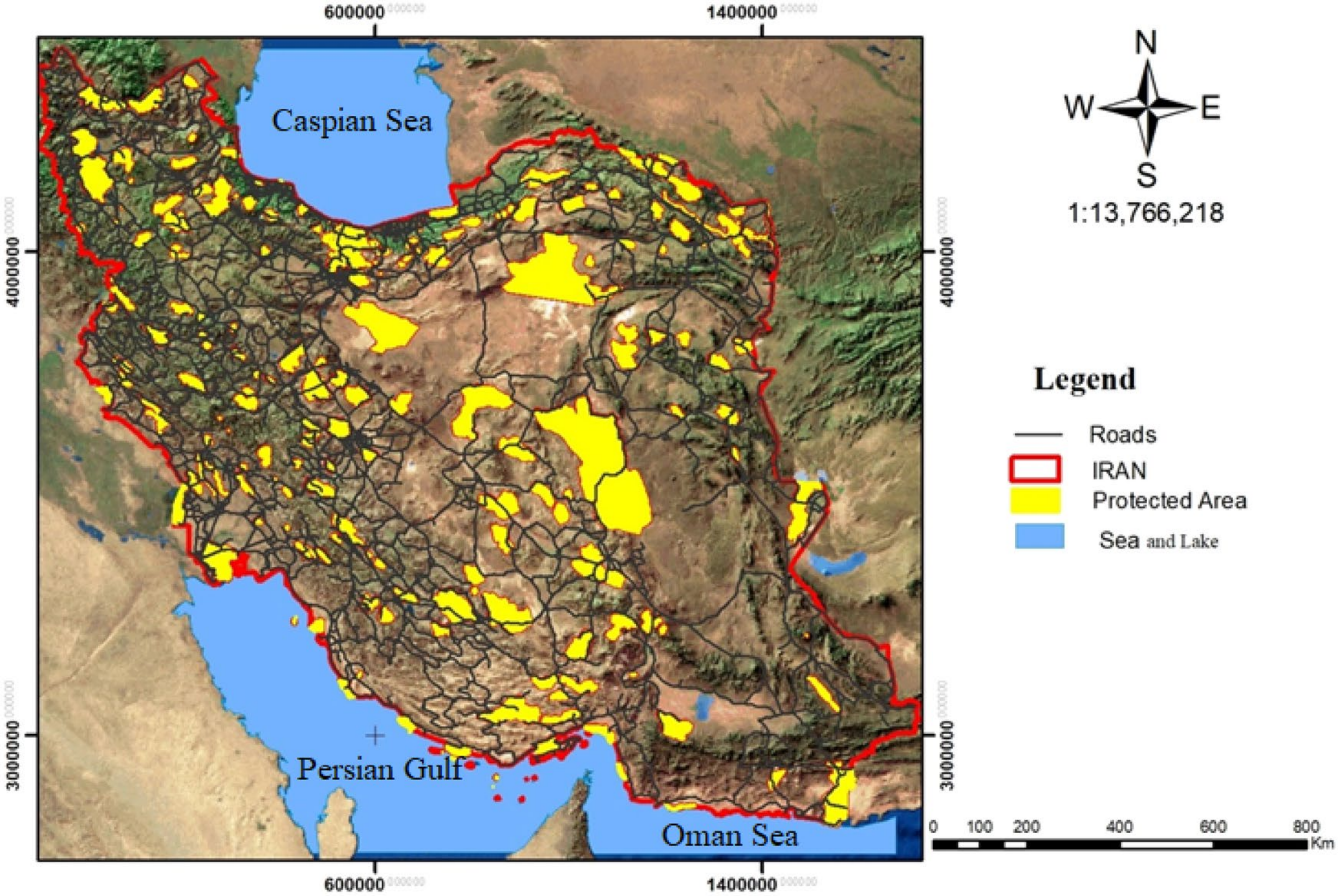
Map of roads and protected areas in Iran using Arcgis 10.3.1 software (URL: https://www.arcgis.com/index.html)^1^.

The study region is the terrestrial biomes of Iran (Fig. 1), located in northeastern Asia, among latitudes 24–40°N and longitudes 44–64°E. With a region of around 164,800,000 ha, Iran is the seventeenth largest country in the world, which includes mountain ranges and highland basins. Iran’s geographical characteristics and vast latitudinal range mean that the climate varies from arid to subtropical throughout the country^19,20^. The altitude range extends from -26 to 5671 m above sea level, the average annual temperature ranges from 8 to 55 °C, the average annual rainfall is 260 mm, the climate is mainly arid and semiarid; except for the northern coastal areas and parts of the western region, the climate is characterized as extremely continental with hot and dry summers and very cold winters, particularly in inland areas. Due to this high geographic variation, a wide variety of flora and fauna inhabit Iran. The eastern part of the country is surrounded by desert, while the northern part is bordered by the East–West trending Alborz mountains, the Caspian Sea and dense forests. The second major mountain range, the Zagros, is located in the country’s western part and extends from north to south. Finally, the southern border of the country is formed by the coastlines of Oman Sea and Persian Gulf^21^. The interplay of the climatic and topographic features in the country plays a major role in the formation of biodiversity. Three eminent realms, including Afrotropic, Palearctic, and Indomalayan also influence the natural characteristics of Iran^22^.

### Data layers

#### Protected areas

Iran’s layer of protected areas in 2017, mapped at a scale of 1: 50,000, was prepared by the Department of Environment (DOE) and included three types: protected areas, national parks, and wildlife refuges^23^. It covers an area of about 16,323,560 ha (Fig. 1).

#### Roads

The layer of Iran’s roads in 2017, mapped at a scale of 1: 50,000, was prepared by the Ministry of Roads & Urban Development (MRUD) and consisted of four types of roads with an overall length of about 103,393 km as followings: highways (20,523 km), main roads (25,866 km), side roads (42,926 km) and railways (14,078km) (Fig. 1)^23^.

#### Terrestrial biomes and important species

**Figure 2:**
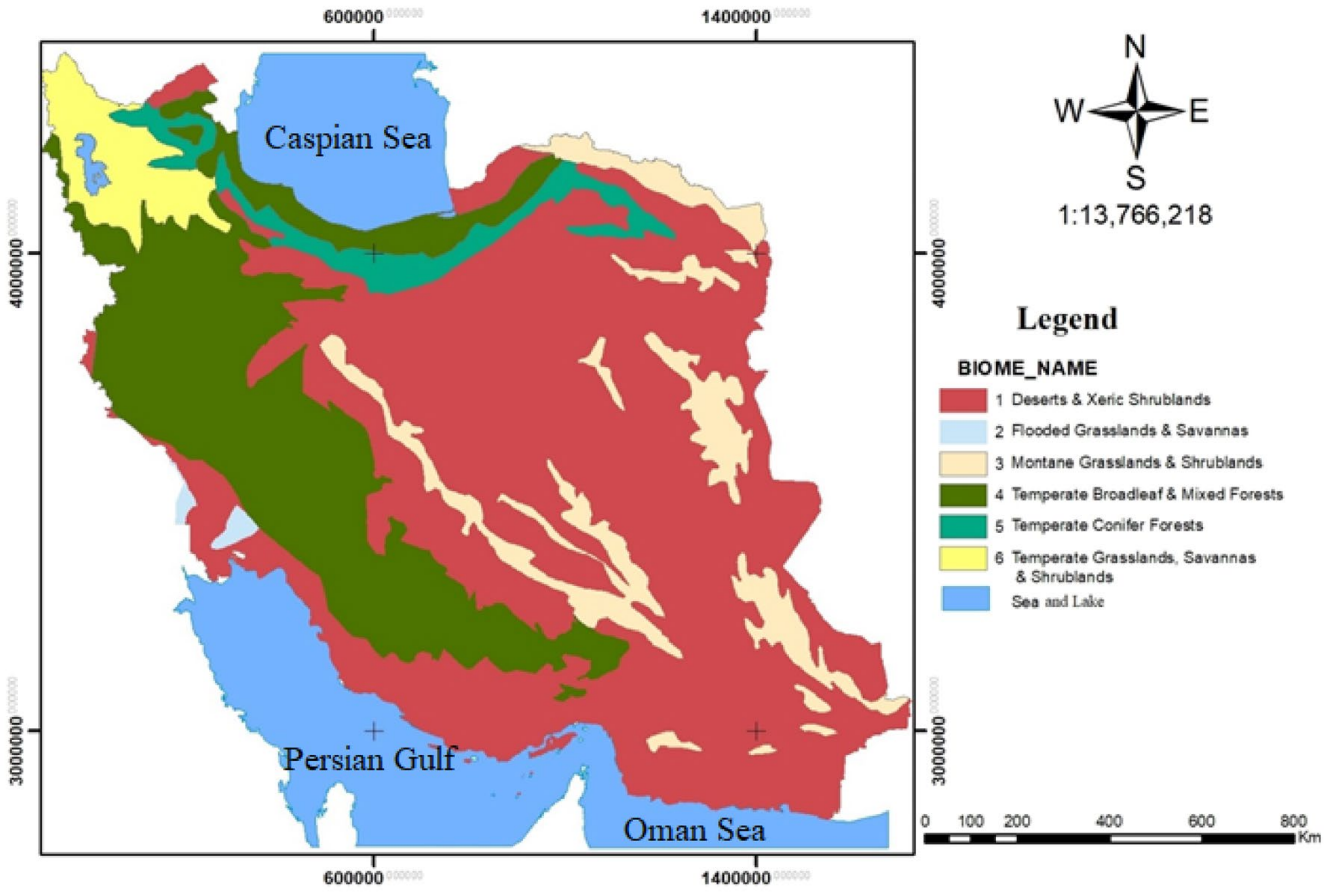
Map of terrestrial biomes in Iran using Arcgis 10.3.1 software (URL: https://www.arcgis.com/index.html)^1^.

**Figure 3:**
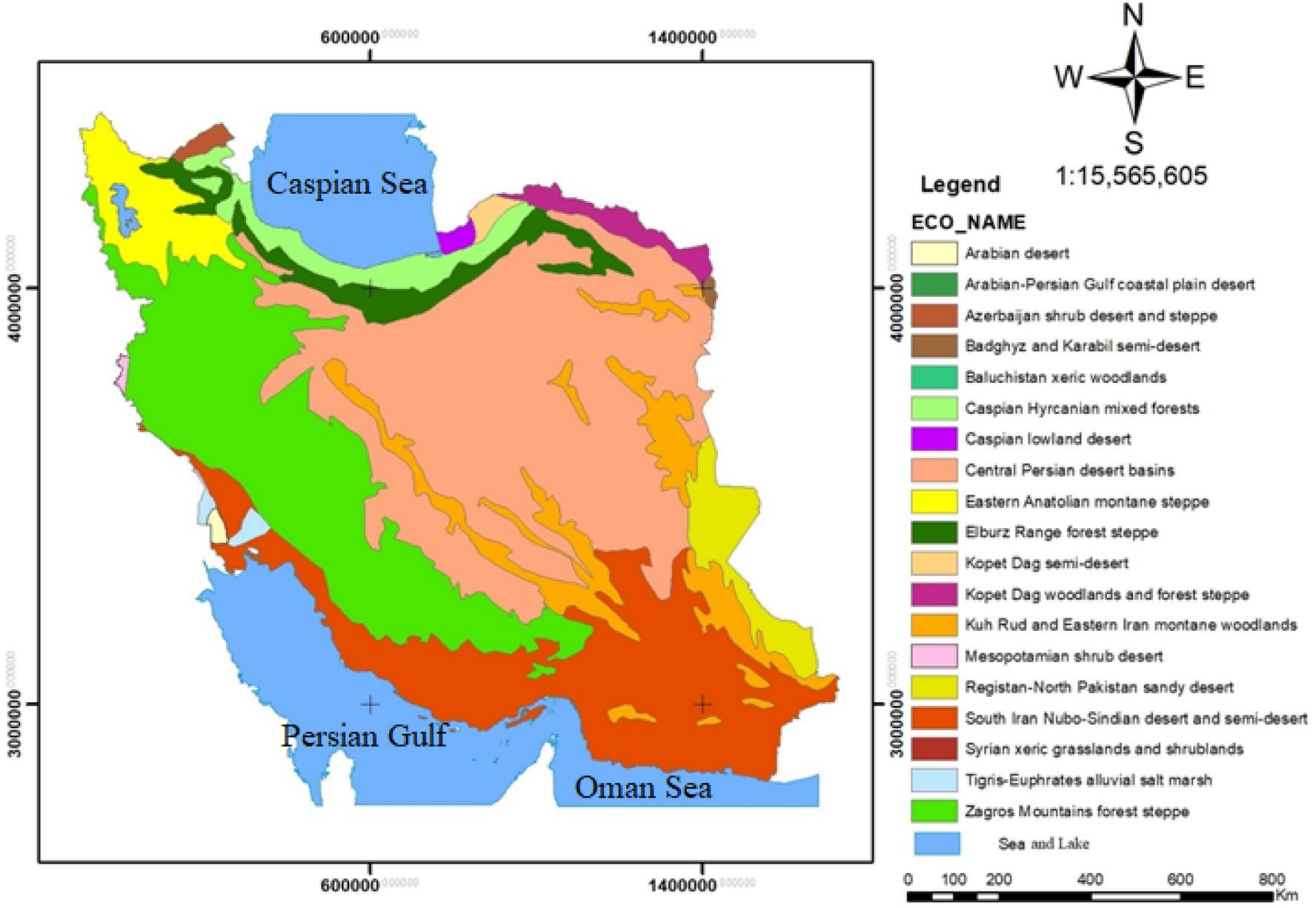
Map of terrestrial ecoregions in Iran using Arcgis 10.3.1 software (URL: https://www.arcgis.com/index.html)^1^.

The types of World Terrestrial Biomes were used to determine the boundaries of terrestrial biomes in Iran^1^. Iran’s layer of terrestrial biomes was mapped at a scale of 1:50,000 and included six terrestrial biomes (Fig. 2) and 19 terrestrial ecoregions (Fig. 3), both with an area of about 162,629,464 ha. Based on the previous reports^1,4,23^, the Iran’s six terrestrial biomes and their representative mammalian fauna are listed in Table 1.

**Table 1:** Terrestrial biomes of Iran and associated representative mammalian fauna.

Terrestrial Biome	Area(ha)	Mammalian fauna
Deserts and xeric shrublands (DXS)	93,962,024	*Acinonyx jubatus, Gazella subgutturosa, Equus onager, Podoces pleskei, Chlamydotis macqueenii and Allactaga firouzi*
Flooded grasslands and savannas (FGS)	720,760	*Dama dama mesopotamica, Crocidura susiana*
Montane grasslands and shrublands (MGS)	14,955,512	*Panther pardus saxicolor, Capra aegagrus aegagrus*
Temperate broadleaf and mixed forest (TBMF)	40,212,864	*Sciurus anomalus*
Temperate coniferous forest (TCF)	6,344,008	*Cervus elaphus maral, Capreolus capreolus*
Temperate grasslands, savannas, and shrublands (TGSS)	162,629,464	*Otis tarda, Ovis orientalis gmelini, Lyrurus mlokosiewiczi*

#### Land cover

**Figure 4:**
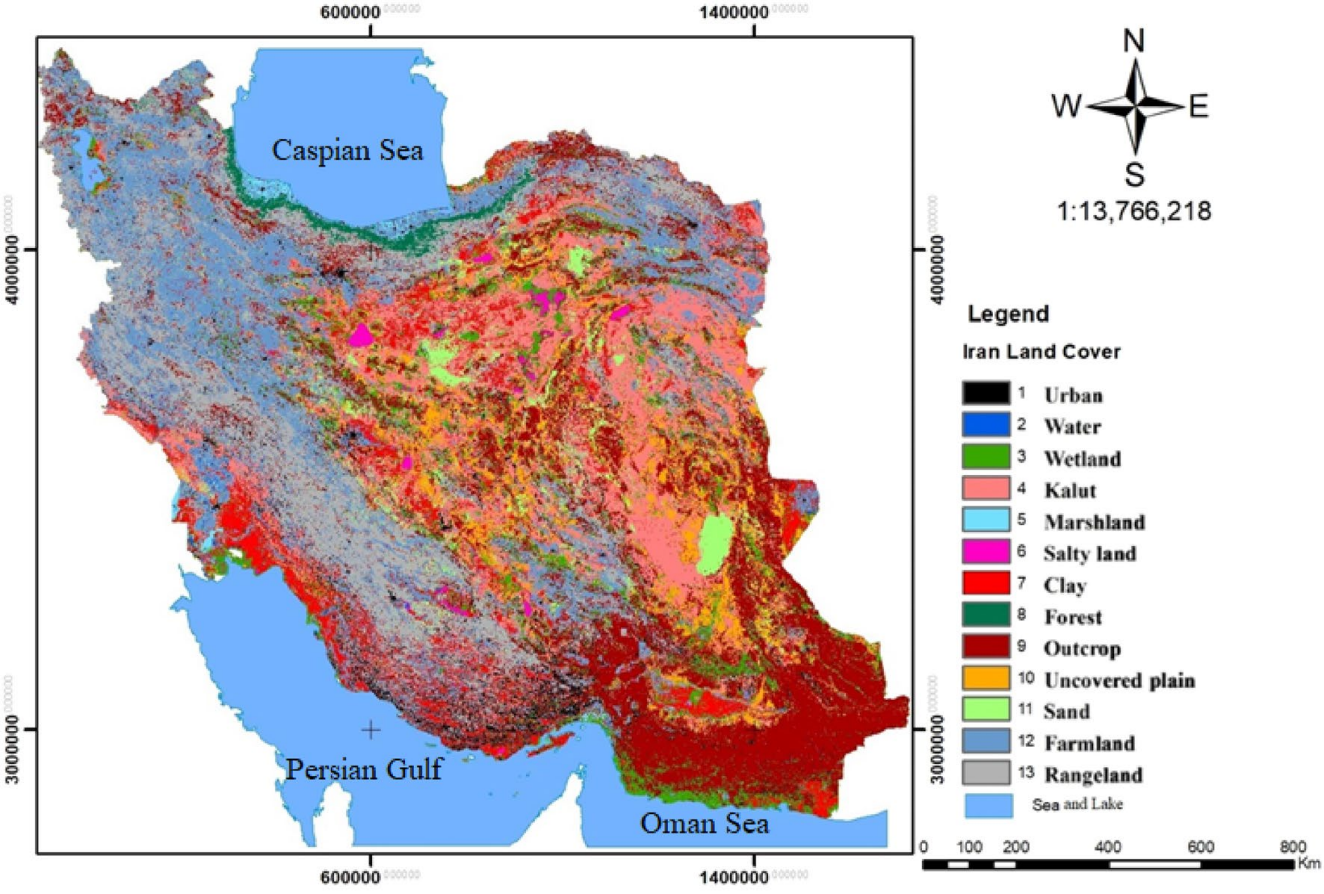
Iran-wide map of land cover types using Terrset 18 software (URL: https://clarklabs.org/terrset/)^21^.

Land cover types of the Iran’s terrestrial biomes were used to determine the boundaries of the Iran-wide land cover maps. The layer of Iran-wide land cover (Fig. 4), mapped at a scale of 1: 50,000, and at included 13 classes of ground cover with an area of about 162,629,464 ha. The land cover types consisted of Rangeland, Kalut desert, Salty land, Clay, Outcrop, Uncovered plain, Sand and rangeland, Farmland, Urban, Wetland, Water, Marshland, and Forest^21^.

### Methods

The IDRISI TerrSet 18.31 software and the Ecosystem Service Modeler (ESM), along with Iran’s 2017 maps of roads, protected areas, terrestrial biomes, and land cover were employed to identify the geographic features used for the present study. We also used variables such as roads, agricultural lands and urban areas (both residential and industrial) for Habitat Risk Assessment (HRA) and Habitat Quality and Rarity (HQR) evaluations^24^. The Terrestrial Biomes Modeler of Ecosystem Service, founded on the toolkit of InVEST (TBESMI) in the Habitat Rarity and Quality model, assesses the impacts of the human threat on the habitats rarity and quality. This is a comprehensive model for evaluating the sensitivity of habitats to changes in the landscape caused by both land threats and land decay.

The results generated by this model permit a rapid evaluation of territorial habitat status, which can serve as a foundation for in-depth research on species adaptability conditions. The table of threats refers the threats that have affected habitats within Iran's terrestrial biomes. The table of sensitivity contains data about habitats and their respective sensitivity to any threat specified in the table of threats. The Habitat Risk Assessment model assesses the risk of man-made activities and management programs for the given habitats. The risk level that a habitat experiences t depends on the degree of exposure to man-made processes and the consequences from this exposure. The model relies on information from maps detailing habitat size and human activities, along with a database that records the intensity and timing of human processes. Additionally, it considers exercises assessing the potential effectiveness of habitat management strategies. Using these inputs, the model creates maps that illustrate the cumulative risk for individual habitats and aggregates the recovery potential across all habitats. The Raster files for terrestrial biomes encompasses stressors and habitats. The total Raster images have a meter-based reference system, including Boolean images. The threats of the Dirt Roads, Agricultural Patchs, and Urban are man-made, which have affected the terrestrial biomes. The distance of maximum (Max_Dist) was used to determine the disturbance zones around the threats of Dirt Road (2 km), Agr_Patch (1 km) and Urban (5 km) within the terrestrial biomes landscape (provided based on field visits and review of sources). The threat weight was 0.8 for Dirt Road, 0.6 for Agr_Patch, and 1.0 for Urban (based on field visits and review of sources). The threat decay was 1.0 for Dirt Road, 0.8 for Agr_Patch, and 1.0 for Urban. The land cover types included Residential, Wetland, Rangeland, Forest, and Cropland. Rangeland, Wetland, and Forest were habitats with a suitability value of 1, while Residential and Cropland were habitats with a suitability value of 0 for Wildlife. The sensitivity value of the Dirt Road threat on Residential and Cropland was 0, on Rangeland was 1, on Wetland was 0.4, and on Forest was 0.8. The sensitivity value of Agr_Patch threat was 0 for Residential and Cropland, 0.8 for Rangeland, and Wetland, and 0.6 for Forest. Finally, the sensitivity value of Urban threat on Rangeland, Wetland, and Forest was 1, whereas it was 0 for Residential and Cropland.

The main threats were those related to agricultural patches, roads, and urban areas. The Tables 1 and 2 display level of sensitivity and threats in the terrestrial biomes. Using HRA and HQR models and data associated with the terrestrial biomes, we assessed the biome threats and sensitivity (Tables 3 and 4), and the the degree of stress in terrestrial biomes (Table 3) (Ronald Eastman 2015). The reliability of maps is based on HQR (Supplementary 1) and HRA (Supplementary 2) working method instructions mentioned in the software. The maps were classified into five classes based on the weight (0–1) in the IDRISI software^25^.

**Table 2:** Terrestrial biomes threats.

Threat	Max_Dist	Weight	Decay
Dirt road	2	0.8	1
Agr_Patch	1	0.6	0.8
Urban	5	1	1

**Table 3:** Terrestrial biomes sensitivity.

Lulc	Name	Habitat	L_dirt_Road	L_agr_patch	L_Urban
1	Residential	0	0	0	0
2	Wetland	1	0.4	0.6	1
3	Rangeland	1	1	0.8	1
4	Forest	1	0.8	0.6	1
5	Cropland	0	0	0	0

**Table 4:** Habitat_stressor_ratings_.

Habitat	Consequence and importance weights						
Habitat ID	Frequency of natural disturbance	Change in structure	Change in area	Regeneration rate	Connectivity	Recruitment pattern	Natural mortality rate
Habitat name	Weight frequency of natural disturbance	Weight change in structure	Weight change in area	Weight regeneration rate	Weight connectivity	Weight recruitment pattern	Weight natural mortality rate

Stressor	Consequence and importance weights						
Stressor ID	Frequency of natural disturbance	Change in structure	Change in area	Regeneration rate	Connectivity	Recruitment pattern	Natural mortality rate
Stressor name	Weight frequency of natural disturbance	Weight change in structure	Weight change in area	Weight regeneration rate	Weight connectivity	Weight recruitment pattern	Weight natural mortality rate

### Ethical approval

This material is the authors own original work, which has not been previously published elsewhere. Also, the manuscript is not currently being considered for publication elsewhere. This manuscript reflects the authors’ own research and analysis in a truthful and complete manner.

### Consent to participate

All authors have been personally and actively involved in substantial work leading to the manuscript and will take public responsibility for its content.

## Results

### Terrestrial biomes of Iran

Table 5 presents the six major terrestrial biomes of Iran and their respective areas. The largest area was DXS (57.77%) and the smallest area was FGS (0.44%).

**Table 5:** Area and percentage of major terrestrial biomes of Iran.

Terrestrial biomes	DXS	FGS	MGS	TBMF	TCF	TGSS	Total
Area (ha)	93,962,024	720,760	14,955,512	40,212,864	6,344,008	643,4296	162,629,464
%	57.77	0.44	9.19	24.73	3.90	3.96	100

### Land cover types

**Figure 5:**
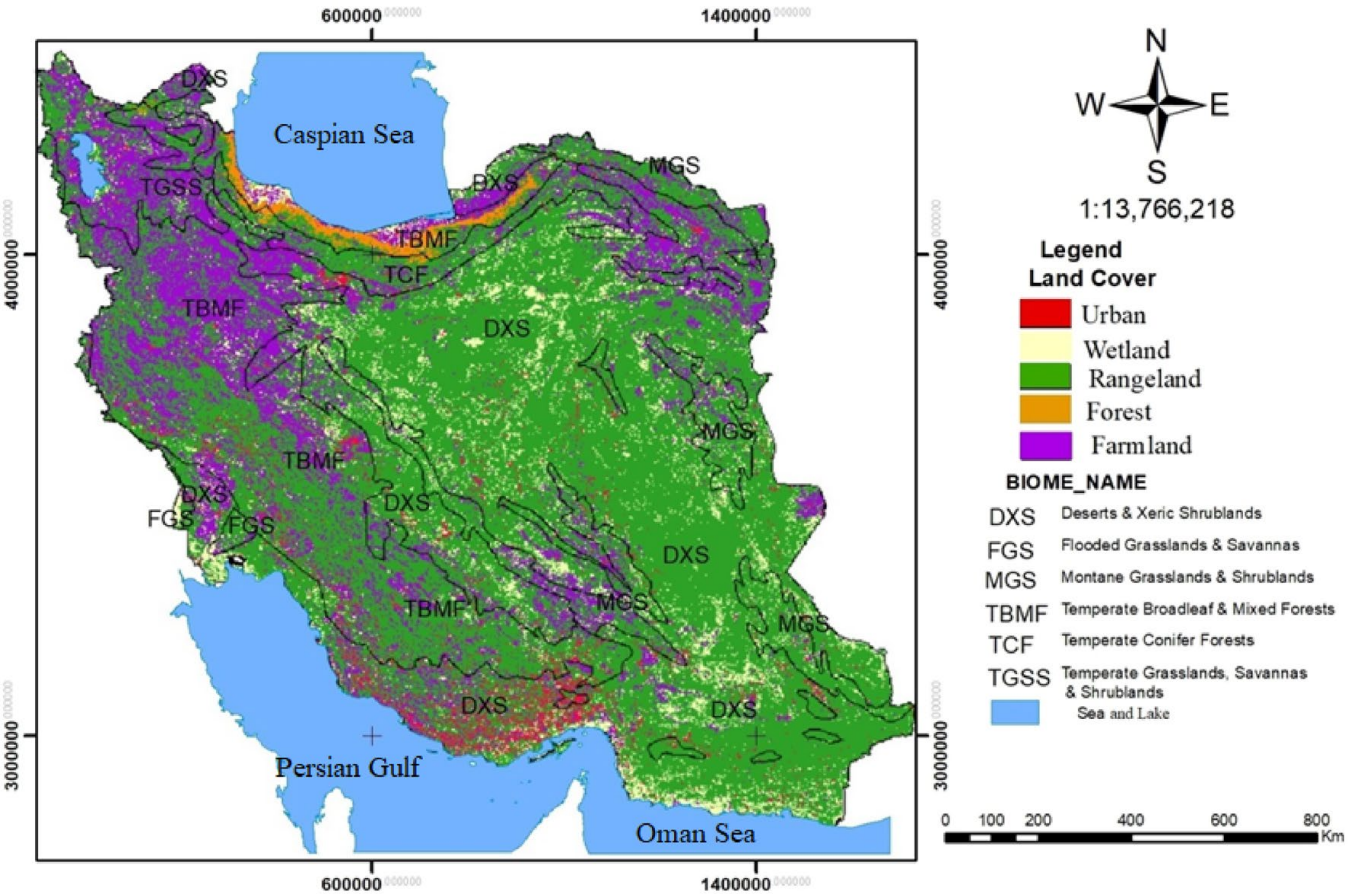
Land cover types in Iran’s terrestrial biomes using Terrset 18 software (URL: https://clarklabs.org/terrset/)^21^.

Five major land cover types were identified in Iran's terrestrial biomes including: Rangeland, Farmland, Urban, Wetland, and Forest. Rangeland included the Kalut Desert, Salty land, Clay, Outcrop, Uncovered plain, Sand, and Rangeland. Wetland included Water, Wetland, and Marshland. Table 6 shows the size of each land cover type and the percentage it represents in each terrestrial biome. This information is also shown graphically in Fig. 5.

**Table 6:** Area and percentage of land cover types in Iran’s terrestrial biomes.

Terrestrial biomes		Urban	Wetland	Rangeland	Forest	Farmland	Total
DXS	Area(ha)	3,243,892	10,037,128	69,819,040	16,040	10,657,484	93,962,024
	%	3.45	10.70	74.30	0.01	11.34	100
FGS	Area(ha)	9028	147,864	447,124	0	111,628	720,760
	%	1.25	20.51	62.03	0	15.49	100
MGS	Area(ha)	244,572	1,296,420	10,955,016	1036	2,444,640	14,955,512
	%	1.63	8.67	73.25	0	16.34	100
TBMF	Area(ha)	1,320,960	1,399,972	22,781,500	1,478,848	13,188,604	40,212,864
	%	3.28	3.48	56.65	3.67	32.80	100
TCF	Area(ha)	88,912	256,516	4,042,132	173,896	1,782,552	6,344,008
	%	1.40	4.04	63.71	2.74	28.01	100
TGSS	Area(ha)	87,520	305,880	2,522,644	11,824	3,489,864	6,434,296
	%	1.36	4.75	39.20	0.18	54.24	100
Total	Area(ha)	4,994,884	13,443,780	110,567,456	1,681,644	31,674,772	162,629,464
	%	3.07	8.26	67.99	1.03	19.47	100

### Quality and rarity assessment of Iran’s terrestrial biomes

**Figure 6:**
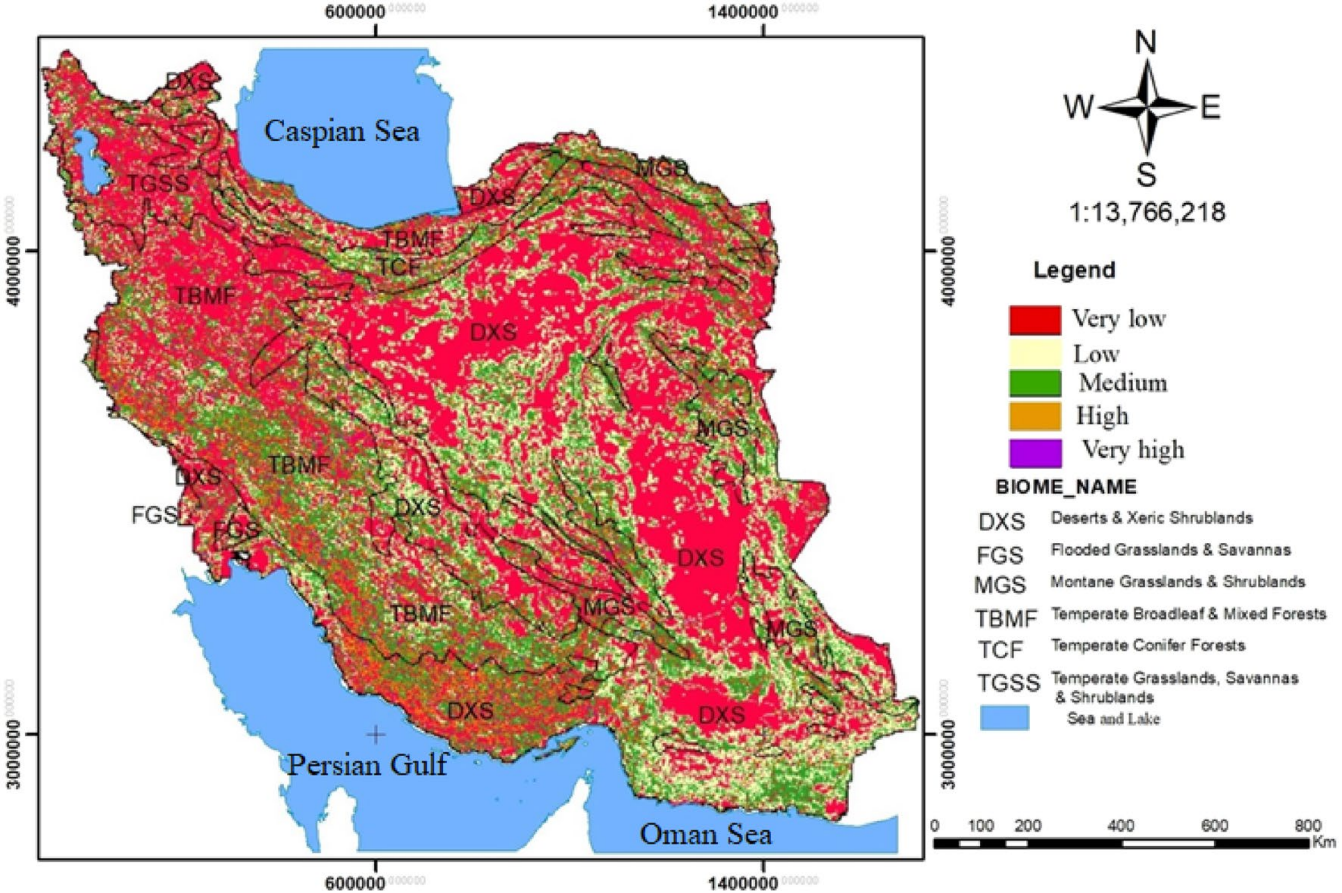
Relative level of habitat degradation on current landscape in Iran’s terrestrial biomes using Terrset 18 software (URL: https://clarklabs.org/terrset/)^21^.

The results presented in Fig. 6 and Table 7 indicate that there was an increase in habitat degradation due to roads, agriculture, and urbanization in the TBMF (11.08%), TCF (8.84%), and FGS (7.77%) biomes while there was a decrease in the DXS (5.14%) biome.

**Table 7:** Area and percentage of habitats degradation in Iran's terrestrial biomes.

Terrestrial biomes		Very low	Low	Medium	High	Very high	Total
DXS	Area(ha)	47,975,980	21,184,408	19,968,420	4,128,820	704,396	93,962,024
	%	51.06	22.54	21.25	4.39	0.75	100
FGS	Area(ha)	351,652	167,204	145,956	44,296	11,652	720,760
	%	48.79	23.20	20.25	6.15	1.62	100
MGS	Area(ha)	5,486,548	3,638,488	4,793,544	901,704	135,228	14,955,512
	%	36.68	24.33	32.05	6.03	0.90	100
TBMF	Area(ha)	18,211,696	5,816,204	11,724,788	3,633,076	827,100	40,212,864
	%	45.29	14.46	2.91	9.03	2.05	100
TCF	Area(ha)	2,586,884	1,210,816	1,985,048	459,072	102,188	6,344,008
	%	40.77	19.08	31.30	7.23	1.61	100
TGSS	Area(ha)	4,198,004	751,456	1,127,152	281,696	75,988	6,434,296
	%	66.17	11.84	17.76	4.44	1.20	100
Total	Area(ha)	78,810,764	32,768,576	39,744,908	9,448,664	1,856,552	162,629,464
	%	48.46	20.15	24.44	5.81	1.41	100

**Figure 7:**
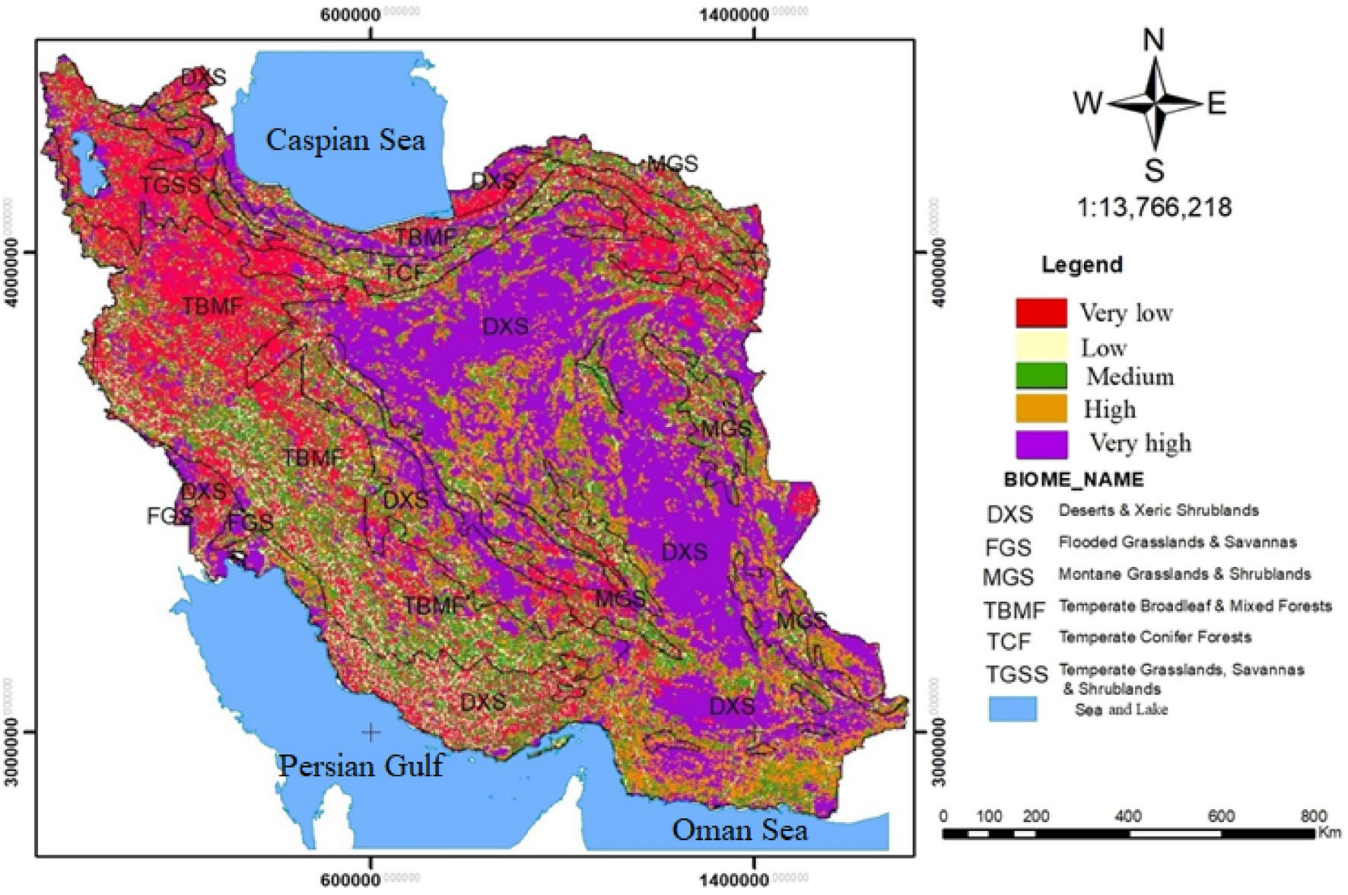
Current landscape habitat quality in Iran’s terrestrial biomes using Terrset 18 software (URL: https://clarklabs.org/terrset/)^21^.

Figure 7 and Table 8 show that there were increases of the quality of habitat landscape in the DXS (62.91%), FGS (57.44%), and MGS (47.44%) biomes and decreases in the quality of habitat landscape in the TGSS (23.19%) and TBMF (26.59%) biomes.

**Table 8:** Area and percentage of habitat quality in Iran’s terrestrial biomes.

Terrestrial biomes		Very low	Low	Medium	High	Very high	Total
DXS	Area(ha)	14,390,476	5,040,808	15,412,192	17,789,040	41,329,508	93,962,024
	%	15.31	5.36	16.40	18.93	43.98	100
FGS	Area(ha)	130,680	56,588	119,404	118,456	295,632	720,760
	%	18.13	7.85	16.56	16.43	41.01	100
MGS	Area(ha)	2,755,180	1,143,516	3,960,908	3,072,448	4,023,460	14,955,512
	%	18.42	7.64	26.48	20.54	26.90	100
TBMF	Area(ha)	14,913,744	4,610,656	9,992,964	5,141,424	5,554,076	40,212,864
	%	37.08	11.46	24.85	12.78	13.81	100
TCF	Area(ha)	1,913,236	595,276	1,684,044	1,021,572	1,129,880	6,344,008
	%	30.16	9.38	26.54	16.10	17.81	100
TGSS	Area(ha)	3,628,328	363,600	949,624	623,104	869,640	6,434,296
	%	56.39	5.65	14.76	9.68	13.51	100
Total	Area(ha)	37,731,644	11,810,444	32,119,136	27,766,044	53,202,196	162,629,464
	%	23.20	7.26	19.75	17.07	32.71	100

### Risk assessment of Iran’s terrestrial biomes

**Figure 8:**
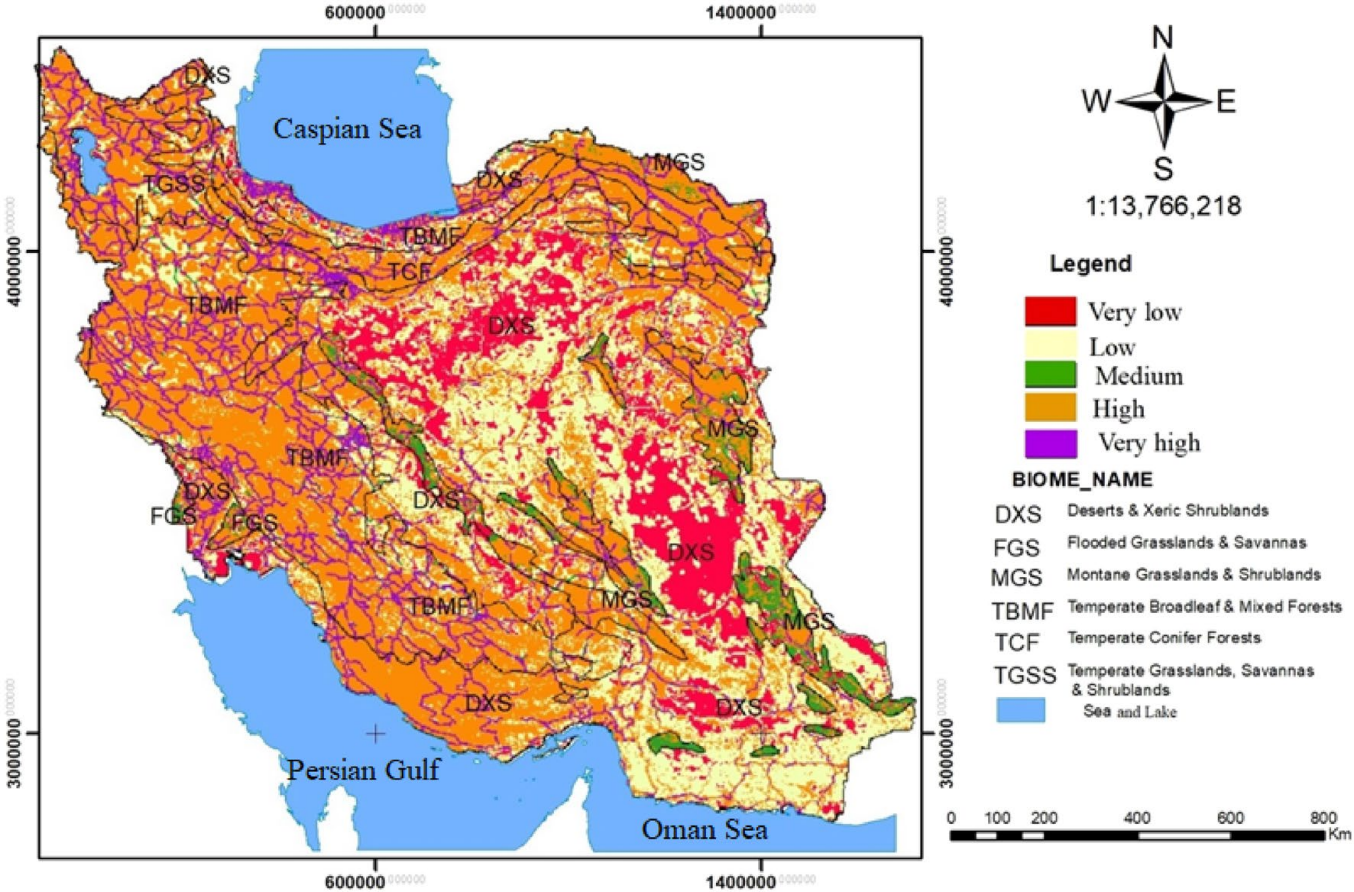
Cumulative risk assessment for all habitats in the Iran’s terrestrial biomes using Terrset 18 software (URL: https://clarklabs.org/terrset/)^21^.

The results showed that there was an increased cumulative risk in the TCF (86.61), TGSS (85.83%), and TBMF (83.1) biomes, and a decreased cumulative risk in the DXS (41.12) biome (Fig. 8 and Table 9).

**Table 9:** Habitat area, percentage, and associated risks in Iran’s terrestrial biomes.

Terrestrial biomes		Very low	Low	Medium	High	Very high	Total
DXS	Area(ha)	19,306,912	35,570,308	446,760	31,600,116	7,037,928	93,962,024
	%	20.54	37.85	0.47	33.63	7.49	100
FGS	Area(ha)	82,944	62,008	150,636	315,500	109,672	720,760
	%	11.51	8.60	20.90	43.77	15.21	100
MGS	Area(ha)	891,028	744,912	3,517,792	8,459,008	1,342,772	14,955,512
	%	5.96	4.98	23.52	56.56	8.96	100
TBMF	Area(ha)	736,536	5,406,316	652,748	25,174,140	8,243,124	40,212,864
	%	1.83	13.44	1.62	62.60	20.50	100
TCF	Area(ha)	131,248	717,780	0	4,484,000	1,010,980	6,344,008
	%	2.06	11.31	0	70.68	15.93	100
TGSS	Area(ha)	109,516	716,272	85,816	4,032,356	1,490,336	6,434,296
	%	1.70	11.13	1.33	62.67	23.16	100
Total	Area(ha)	21,258,184	43,217,596	4,853,752	74,065,120	19,234,812	162,629,464
	%	13.07	26.57	2.98	45.54	11.83	100

### Protected areas

Table 10 shows an increase in the quality of habitat landscape in the protected areas (57.95%). There was a decrease in the habitat degradation in the Road, Agriculture, and Urban areas located within the protected areas (5.56%) and an increase in the cumulative risk in the protected areas (48.67%).

**Table 10:** Area and percentage of protected areas in Iran’s terrestrial biomes.

Habitat variables		Very low	Low	Medium	High	Very high	Total
Quality	Area(ha)	2,325,372	992,856	3,545,740	3,114,308	6,345,284	16,323,560
	%	14.24	6.08	21.72	19.08	38.87	100
Degradation	Area(ha)	7,364,320	3,651,220	4,398,788	807,284	101,948	16,323,560
	%	45.11	22.37	26.94	4.94	0.62	100
Risk	Area(ha)	2,714,984	5,392,604	270,696	6,989,420	955,856	16,323,560
	%	16.63	33.03	1.66	42.82	5.85	100

**Figure 9:**
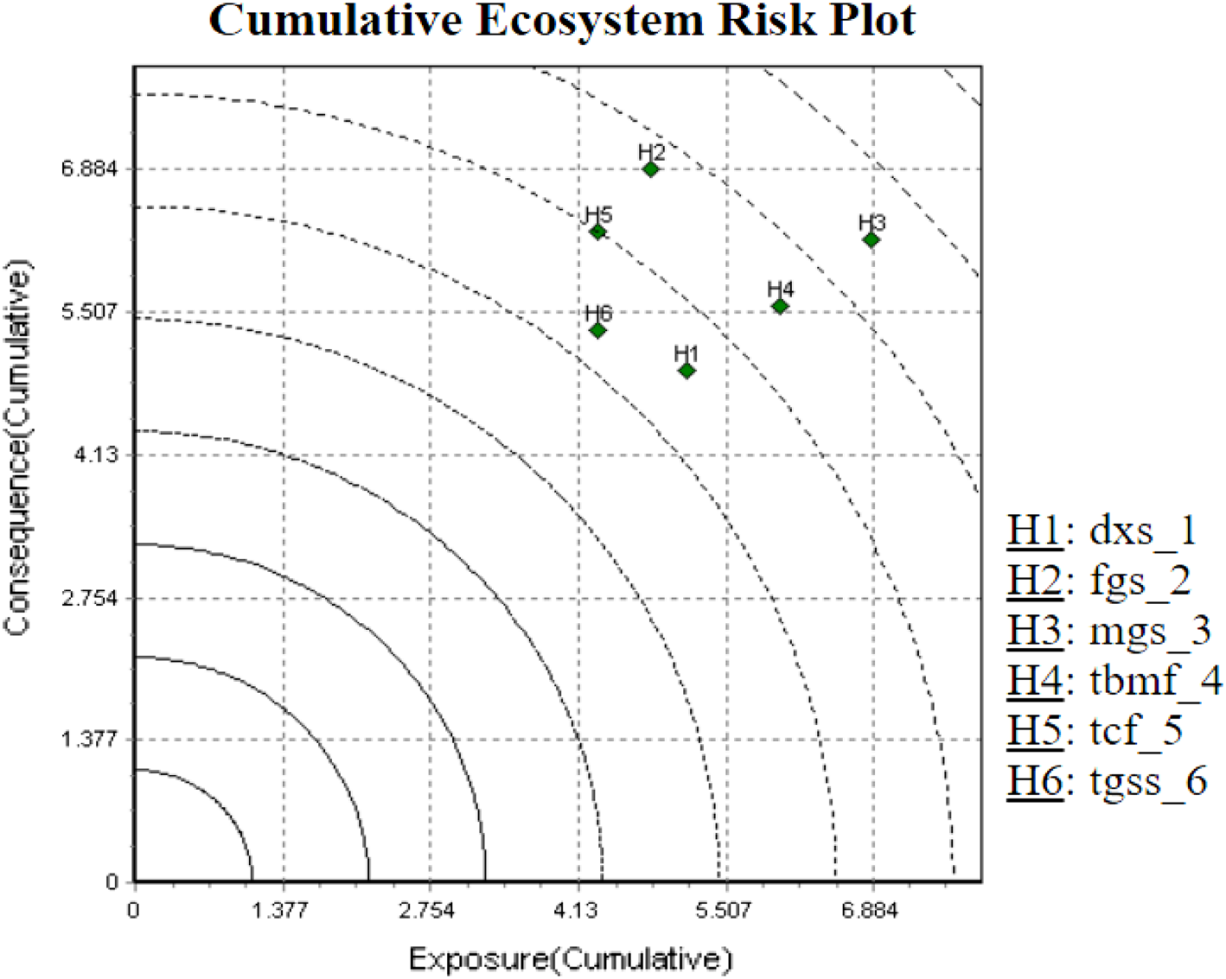
Cumulative ecosystem risk plot of Iran’s terrestrial biomes.

Figure 9 shows the cumulative risk for each terrestrial biome of Iran. This plot can be utilized to demonstrate that terrestrial biomes are at the highest risk due to man-made activities. This risk may result from a cumulative high exposure to external factors that can be mitigated through management or from the outcomes of a high cumulative effect caused by internal factors that are less responsive to human intervention. Cumulative exposure scores were calculated by summing the exposure scores for each stressor in the study area. Cumulative consequences scores were created in the same way. Terrestrial biomes with high cumulative consequences scores and high cumulative exposure scores are at the highest risk related to human activities. Figure 9 displays the cumulative risks for FGS, TCF, and MGS terrestrial biomes in Iran. DXS with a low cumulative consequence score, and TCF and TGSS with low cumulative exposure scores are at the lowest risk arising from man-made activities. FGS terrestrial biome with a high cumulative consequence score and MGS with a high cumulative exposure score are at the highest risk from human activities.

**Figure 10:**
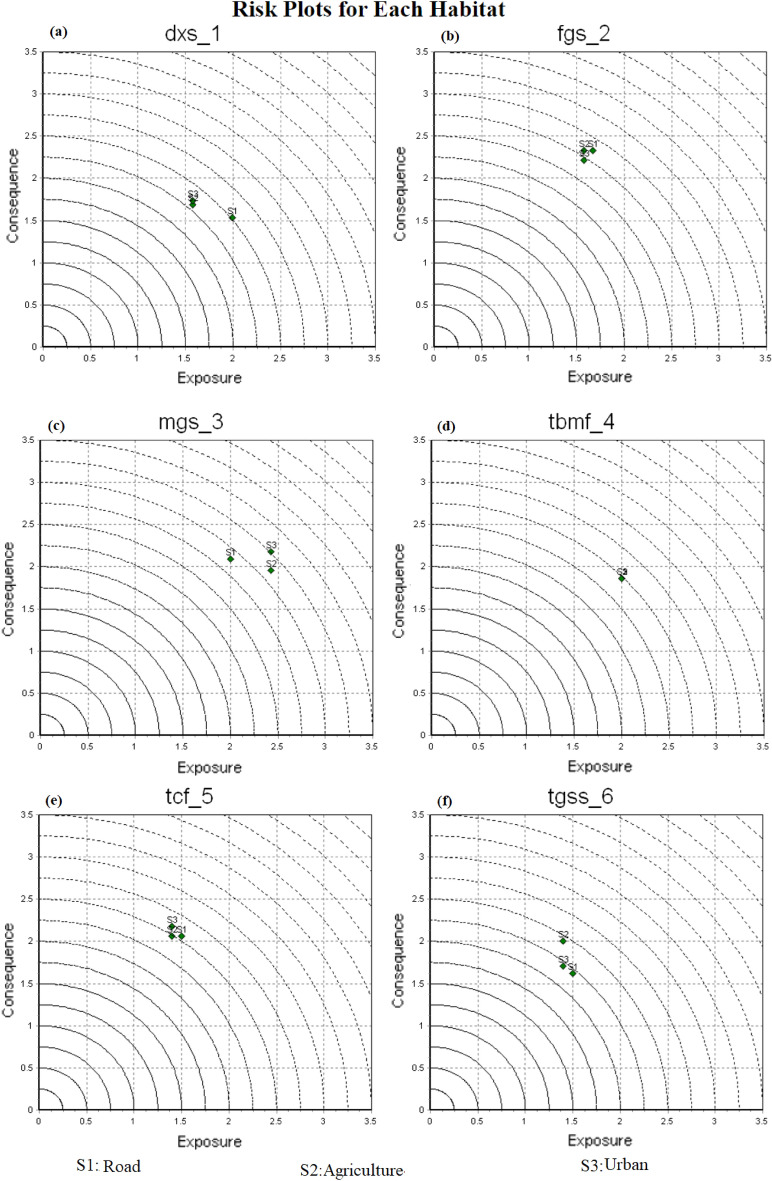
Risk plots for Iran’s terrestrial biomes.

Consequence and exposure scores corresponding to each stressor (Agriculture, Road, and Urban) and terrestrial biome are presented in Fig. 10. Stressors with high exposure scores and high consequence scores pose the greatest risk to the respective terrestrial biome. The effectiveness of risk reduction through management strategies is likely to be more efficient in situations where the risk primarily arises from high exposure, rather than high consequences.

According to the results, stressors with high exposure scores caused the greatest risk to the MGS biome, whereas stressors with high consequence scores caused the greatest risk to the FGS biome. Urban and Agriculture stressors had high exposure scores in the MGS biome, while Roads had high exposure scores in the TBMF and DXS biomes. Stressors of Roads, Agriculture, and Urban had high consequence scores in the FGS terrestrial biome.

## Discussion

The Terrestrial Biomes Modeler of Service, built upon the InVEST toolkit (TBESMI), has applied the concept of 'habitat-impact of source' risk pathways to the large-scale Iran’s terrestrial biomes region. Within the framework of a Relative Risk Model (RRM), TBESMI specifically addresses environmental risks, which is deeply concerned with detrimental stressors and their consequences on vulnerable habitats. TBESMI facilitates assessments of risk at the terrestrial biome scale, taking into account complex interactions among multiple sources, habitats, and cascading effects. Furthermore, TBESMI has enhanced the source (stressor) ranking process by employing multiple criteria that leverage the available source information. This approach is capable of providing quantitative data to identify the most significant sources of risk within terrestrial biomes, thereby improving the assessment accuracy.

We evaluated the cumulative risks posed to the terrestrial biomes patches by man-made activities and explored options for biodiversity and ecosystem services delivery using InVEST in order to project levels of patch fragmentation into the future. The HRA, which employs a framework of exposure consequence to evaluate spatial variations in the cumulative risk of multiple man-made activities such as land cover change, agriculture, roads and urban landscape, was also utilized in our analysis. This was an important factor to be included as the cumulative risk evaluation provides an analytical framework for combining the impact of multiple man-made stressors across the terrestrial biomes, supporting decision-making to balance man-made activities and ecosystem health^26^.

Our findings, based on accurately estimated values of maximum distance (Max_Dist), weight, and sensitivity for defining disturbance zones, revealed that Dirt Road, Agr_Patch, and Urban landscape are the greatest threats to wildlife in the Forest, Wetland, Rangeland, Cropland, and Residential terrestrial biomes. DXS is the largest area among the Iran’s terrestrial biomes, while the FGS is the smallest. There was an increase in habitat destruction in the road, agriculture, and urban areas around the TBMF, TCF, FGS, while it decreased in the DXS biome. There was an increase in the quality of landscape habitat in the DXS, FGS, and MGS, while the quality decreased in the TGSS and TBMF biomes. These results are in line with previous studies regarding the cascading effects^25,27,28^.

There was an increase in the quality of landscape habitat in the protected areas, but a decrease in the Road, Agriculture, and man-made landscapes around the protected area. Thus, there is an increased cumulative risk in the protected areas. This is in agreement with findings of other studies^29^ that demonstrated great variations in cumulative risks within Iran's biomes with different protected area categories. Given that any class of the protected area has distinct goals, and the existence of all categories is necessary to achieve the biodiversity conservation objectives, it is recommended than, in the future development of protected areas in the country, appropriate coverage for each biome should be discussed. This means that a comprehensive country's biomes map should be the basis for selecting new protected areas. These discoveries align with the outcomes of other studies in Iran^30,31^, where it was found that topography and food availability in the region were the main variables defining the suitability of habitat for *Ovis orientalis* (a species of wild sheep). Likewise, land cover and distance from human activities and roads were found to be the most significant variables underlying the suitability of habitats for big mammal species^32–36^. There was an increased cumulative risk in the TCF, TGSS, TBMF biomes and a decreased cumulative risk in the DXS biome. . Habitat degradation and fragmentation, land use changes, road deaths, illegal hunting, periodical droughts, and the prevalence of diseases are the major threat factors for wildlife in Iran^1,23^.

Our findings also indicate that the most stressful factor was related to agricultural lands, in line with other studies^4,28,37–40^. The highest increased cumulative risks belonged to the FGS, TCF, and MGS biomes. DXS biome with a low cumulative consequence, and TCF and TGSS biomes with low cumulative exposure were at the highest risk from human activities. FGS biome with high cumulative consequences and MGS with high cumulative exposure were found to be at the highest risk related to man-made activities. The assumption that variability in mutability is influenced by other factors, including biodiversity, is previously supported by researchers^41,42^. Due to high demands driven by man-made activities^43^, protected areas are critical for the survival of species and protection of threatened ecosystems facing changes in land use and habitat loss^44^. Stressors with high exposure scores caused the greatest risk to MGS biome. Stressors with high consequence scores caused the greatest risk to FGS biome. Urban and Agriculture stressors had high exposure scores in the MGS biome, whereas Roads had high exposure scores in the TBMF and DXS biomes. The stressors of Roads, Agriculture, and Urban had high consequence scores in the FGS biome. Our findings are in agreement with previous studies^2–4,38–40^, which emphasized that the biodiversity in Iran is threatened by various factors including population growth, increased man-made activities, drought, climate change, agriculture, desertification, political conflicts, and economic sanctions.

Biodiversity hotspots for endangered mammal species in Iran are concentrated in the western, central, and northern regions, particularly in areas along the Alborz and Zagros mountains. However, habitats for these threatened mammal species are confined to small areas within Iran, which covers only about 27% of the country's landmass. These regions suffer from severe fragmentation, with only 57% of them currently designated as protected areas under the existing conservation programs. Therefore, it is recommended that conservation efforts focus on these biodiversity hotspots that are not officially protected by conservation laws. The construction of roads and deforestation, driven by urban and village developments as well as wood harvesting, poses a specific threat to the populations of large mammals. Over the past five decades, eastern Iran has served as a last refuge for surviving Asiatic Cheetahs. Roads disrupt the integrity of forests, making them accessible to illegal hunters and leading to road-kill mortality. The primary threat to mammals in the Caspian forests is habitat degradation and loss, particularly impacting large herbivores and the Brown bear. Agricultural cultivation and overgrazing further contribute to the reduction of natural vegetation in the Zagros Mountains forest. Key threats to biodiversity encompass water scarcity, land degradation, pollution, and dust^23^.

Man-made activities leading to fragmentation and habitat loss are the greatest threats to biodiversity vulnerabilities^45–48^. Mountain grasslands are highly vulnerable to changes in land use^49,50^, and tend to lose a significant portion of their primary expanse, leading to increased fragmentation due to man-made stressors^51^. Identifying suitable habitats and evaluating connectivity are crucial for maintaining key landscapes. Fragmented habitats can be caused by biotic and abiotic factors that produce patchiness in natural landscapes, and human disturbances quickly accelerate and intensify habitat fragmentation globally^51^. The protection state of the Iran’s terrestrial biomes is ‘Nature Could Reach Half Protected’ for DXS; ‘Nature Could Recover’ for FGS and MGS; and ‘Nature Imperiled’ for TBMF, TCF, and TGSS^1,3^.

## Conclusions and future prospects

The TBESMI toolkit facilitates comprehensive risk assessment at the terrestrial biome scale. Within the Iran’s terrestrial biome, habitats for fauna species have become fragmented into various patches due to human-related factors. In the DXS biome, we observed improved habitat conditions with reduced degradation and lower cumulative risk. Conversely, the presence of low cumulative risk consequences and the influence of Road-related stressors contributed to elevated exposure scores. The TBMF biome experiences heightened habitat degradation and reduced landscape quality, leading to increased cumulative risk. Here, Roads also play a significant role in elevating the exposure scores. The FGS biome exhibits a rise in habitat degradation and cumulative risk, primarily driven by stressors from Agriculture and Urban areas, resulting in higher consequence scores. Meanwhile, the TCF biome faces amplified habitat degradation, leading to an overall increase in cumulative risk. Stressors in this biome contributed to elevated consequence scores. In the MGS biome, we observed improved habitat landscape quality along with an increased cumulative risk. Stressors from Agriculture and Urban areas pose high exposure scores and present the greatest risk. Finally, the TGSS biome experiences a decline in habitat landscape quality, coupled with an increase in cumulative risk.

We, accordingly, suggest the following strategies/recommendations for habitat protection and wildlife conservation: (i) Establishing a comprehensive management plan for Iran's terrestrial biomes is imperative; (ii) Mitigating land use changes and the destruction of habitats in the TGSS and DXS terrestrial biomes should be prioritized; (iii) The creation of protected areas within the MGS biome is essential; (iv) National parks should be developed in the MGS, TBMF, and DXS biomes; (v) Conducting a comprehensive risk assessment of Iran's terrestrial ecoregions is crucial; (vi) Efforts to reduce the impacts of Agriculture and Urban activities in the MGS biome should be implemented; (vii) Initiatives to minimize the impacts of Roads in the TBMF biome should be pursued; (viii) Utilizing the TBESMI model to conduct risk assessments of terrestrial biomes in other parts of the world and comparing them with Iran's situation is advisable; (ix) Strategies to mitigate the impacts of cumulative risk in the TBMF, FGS, and MGS biomes are necessary.

### Supplementary Information

Supplementary Information 1.

Supplementary Information 2.

## Data Availability

All data generated or analyzed during this study are included in this published article.
