# Supplementary material for: Ecological assessment of Iran’s terrestrial biomes for wildlife conservation
Source: Sci Rep. 2023 Oct 18;13:17761. doi: 10.1038/s41598-023-45120-4 (PMC10584875; doi:10.1038/s41598-023-45120-4)
Supplement: Supplementary file 1 — Supplementary Information 1. [file 41598_2023_45120_MOESM1_ESM.pdf]

Habitat Quality and Rarity - Ecosystem Services Modeler

The Habitat Quality and Rarity model assess the impacts of anthropogenic threats on the quality and rarity of habitats. It is a generalized model to assess the sensitivity of habitats due to landscape changes both through land degradation and threats to the land. Habitat quality is assessed through land cover change proximity and habitat rarity by looking at the reduction of rare land cover from an historical baseline. The results from the model allow for the rapid assessment of regional habitat conditions that can be used as a proxy for more in-depth investigations into the status of species fitness. The model produces habitat quality and rarity maps that show habitat change across time.

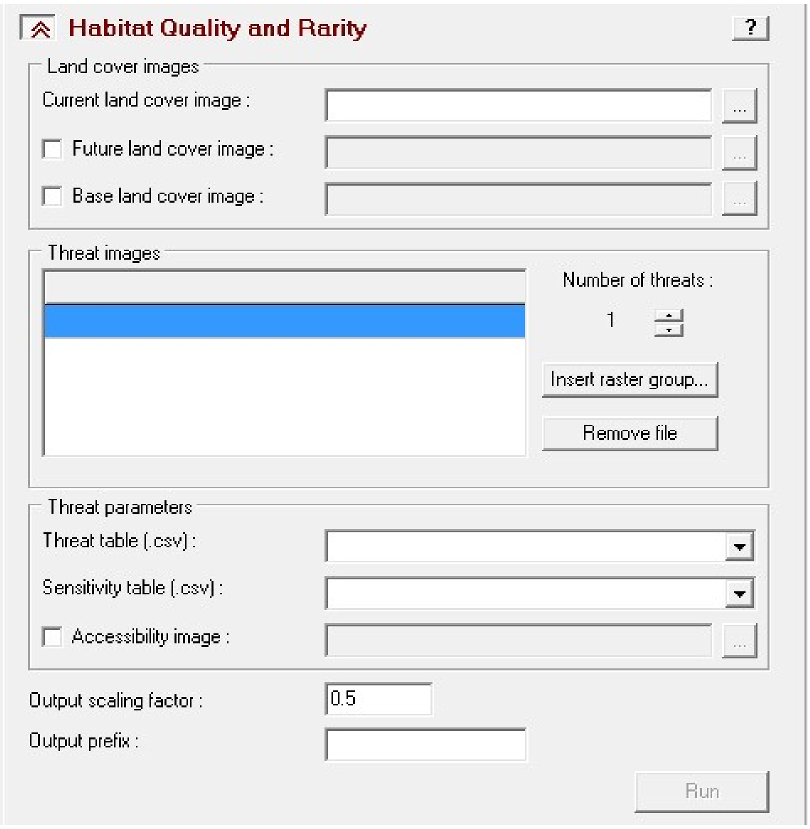

Habitat Quality and Rarity Operation

1. Enter a current land cover map. (See Note 1.)

2. Select to include a future land cover map.

3. Select to include a baseline land cover map.

4. Indicate the number of threats to model, and enter each image in the threat image grid. Alternatively, you can insert a raster group file (.rgf) of threat images. (See Note 2.)

5. Use the drop-down menu to select the threat table (.CSV) containing threat importance weights and distance impact parameters. (See Note 3.)

6. Use the drop-down menu to select the sensitivity table (.CSV) containing the sensitivity of each habitat. (See Note 4.)

7. Select to include an access image of relative protection status across the landscape. (See Note 5.)

8. Specify the output scaling factor. (See Note 6.)

9. Enter the output prefix for the Habitat Quality and Rarity outputs.

10. Click Run.

Notes

1. The land cover map must contain discrete classes with unique integer identifiers and an associated legend for each land cover type. Baseline and future land cover maps are optional. The legends and land cover codes of all input land cover maps must match exactly, even for classes that do not exist in each map. If possible, the baseline land cover map image should be a classification of the study area region that exhibits a landscape prior to the impact of anthropogenic land conversion.

2. Each threat image name is composed of two parts. The first part must be the same name of the threat used in the threat table (see Note 3), followed by a suffix denoting whether the threat is for the current, future or baseline period. For example, using the threat named "dirt_road" from Note 3, the threat map names for the current, future and baseline periods would be, respectively, "dirt_road_c", "dirt_road_f" and "dirt_road_b"

For each threat image, values range from 0 to 1 to indicate the degree of threat intensity (e.g., as defined by density of urban area, population density, proximity to roads, etc.) or a simple Boolean image of presence (1) or absence (0) of a given threat. Note that all threat images must use the same scale – e.g., if one threat is Boolean, all threat maps must be Boolean. Additionally, areas considered to be in no-threat must be given a value of 0.

3. The threat table is a .CSV file with four columns. The four columns must be titled: threat, max_dist, weight, and decay.

Threat - The names of the threats that will impact habitat. Do not use spaces. This name must match the name of the corresponding threat map (without the "_c","_f", or "_ b" suffixes,see Note 2).

Max_Dist - The distance used to define disturbance zones surrounding each threat in the landscape (kilometers).

Weight - The weight applied to each variable as the relative impact per threat. Each threat is designated a real number weight ranging from 0 to 1, where values closer to 1 indicate a high-impact threat and values closer to 0 indicate a lower-impact threat.

Decay - A Boolean indicator to specify whether the distance function will be treated linearly or exponentially across space. A value of 0 indicates that the threat level will decrease exponentially, and a value of 1 will treat the distance function as a linear model.

threat	max_dist	weight	decay
Dirt_Road	2	0.11	0
Agr_Patch	8	.78	1
RailRd	5	.05	0
Urban	6	.64	0

Although the use of future and baseline maps produces a multi-scenario analysis, the impact weights are assumed to remain constant. You can alter weight values if the threat changes over time by running the model more than once for each scenario, treating each scenario as a “current” land cover map.

4. The Sensitivity table contains information on habitats and their relative sensitivity to each threat defined in the threats table. The column headings must be named: *LULC, Name, Habitat, L_<Threat Name1>, L_<Threat Name 2>….L_<Threat Name(n)>.* The prefix "L_" must accompany each threat. For example:

lulc	name	habitat	l_dirt_rd	l_agr_patch	l_railr	l_urban
1	Water	1	0.12	0.2	0.3	0.68
2	Forest	0	0	0	0	0
3	Residential	0	0	0	0	0
4	Beach	1	0.72	0.25	0.3	0.55
6	Cropland	0	0	0	0	0
7	Transportation	0	0	0	0	0
8	Openland	1	0.36	0.12	0.55	1

LULC - The unique integer ID of each land cover type associated with the input land cover maps.

Name - The associated LULC legend name for each land cover type associated with the input land cover maps.

Habitat - The habitat suitability score. A value of 1 indicates that the land cover type is a verified habitat for the species of interest. If it is not a habitat, give it a value of 0. If it is suitable but not ideal, give it a value between 0 and 1 that reflects its suitability.

L_<Threat Name1>, L_<Threat Name 2>….L_<Threat Name(n)> - For each land cover type, values range from 0 to 1 where values close to 1 indicate high sensitivity to an imposing threat and values close to 0 indicate low sensitivity. Regardless if the land cover type is a habitat or not, the cells in the table must not be left blank; instead enter a value of zero.

5. The access image is an optional, Boolean raster image indicating areas accessible to threats (=1) and conservation areas or areas protected from threats (=0).

6. The output scaling factor is used to scale the habitat quality values. This parameter should normally be set to 0.5, indicating that the middle quality rating (0.5) corresponds with an intermediate level of degradation (a position half way between 0 and the maximum degradation).
