# Supplementary material for: Ecological assessment of Iran’s terrestrial biomes for wildlife conservation
Source: Sci Rep. 2023 Oct 18;13:17761. doi: 10.1038/s41598-023-45120-4 (PMC10584875; doi:10.1038/s41598-023-45120-4)
Supplement: Supplementary file 2 — Supplementary Information 2. [file 41598_2023_45120_MOESM2_ESM.pdf]

Habitat Risk Assessment - Ecosystem Services Modeler

The Habitat Risk Assessment model evaluates the risk of human activities and management plans on habitats. The level of risk experienced by a habitat depends on the degree of exposure of the habitat to human activities and the consequences that result from this exposure. The model uses map data on habitat extent and human activities as well as a database on the timing and intensity of the human activity and the potential effectiveness of habitat management practices to output the cumulative risk maps for each habitat and a combined total risk recovery potential for of all habitats.

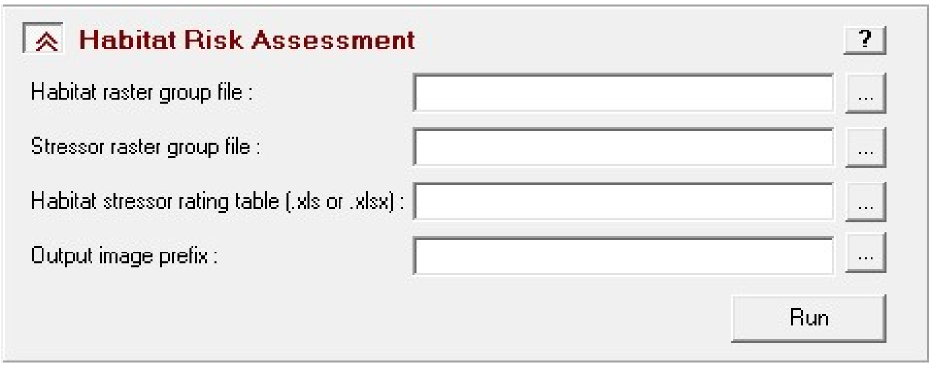

Habitat Risk Assessment Operation

1. Enter the raster group file (.rgf) containing Boolean images of habitats (See Note 1).

2. Enter the raster group file (.rgf) containing Boolean images of human activities (stressors) that impact habitats (See Note 1).

3. Enter the habitat stressor rating table (See Note 2).

4. Enter an output prefix. (See Note 3.)

5. Click Run.

Notes

1. The raster group files for both habitats and stressors is limited to 10 habitats. All raster images must have a reference system specified in meters and be Boolean images.

For the habitat files, the follow the naming convention must be used for each habitat map: “<habitat name>_<habitat ID>.rst”, e.g., “coralreef_1.rst, seagrass_2.rst”. The Habitat ID must correspond to the habitat ID used in the habitat stressor rating table. See Note below.

For the stressor files, the follow the naming convention must be used for each habitat map: “<stressor name>_<stressor ID>.rst”, e.g., “finfish_1.rst, scubadiving_2.rst”. The Stressor ID must correspond to the stressor ID used in the habitat stressor rating table. See Note below.

The habitats included in the raster group file can be associated with a broad array of organisms or they could be limited to one specific species. If you choose to include multiple species in one habitat image, it is important that the species share certain characteristics in their response to stress. For each habitat, it is assumed that there is no diversity in the response to specific human activities. Background or areas that should not be

considered in the analysis should have a value of zero.

2. The Habitat Stressor Rating Table is a Microsoft Excel spreadsheet file (.XLS) that contains much of the information required to run the model. This file must be located in the same folder as your data. A sample file can be found in the Resfiles folder under the TerrSet installation. This sample file is the same one used for the tutorial exercise. You can modify the worksheet to accommodate any type of habitat and stressors. Please backup the spreadsheet before modifying.

The spreadsheet contains three worksheets that must be filled out, although not every row and column in the spreadsheet is necessary. You may enter up to 10 habitats and 10 stressors. You may weigh the data according to their importance in the analysis. Below is a description of the different worksheets.

**Worksheet (1)**	
Column A: Stressor ID	Unique integer IDs used to link each stressor image to its corresponding image.
Column B: List of Stressors	Names of the stressors and a radio button indicating the weight for each stressor.
Column C: Intensity	Intensity level for each stressor with choices of : high intensity, medium intensity, low intensity, or no score (excludes from assessment).
Column D: Management	Importance weight of the management practice to minimize impact of the stressor with choices of: very effective, somewhat effective, not effective or poorly managed, or no score (excludes from assessment).
Column E: Stress Buffer Distance	Distance (m) around each stressor location over which the stressor has an impact. This field is especially useful if there is minimal locational data on stressors. You may enter 0 here if a buffer is unnecessary.

**Worksheet (2)**	
Column A: Habitat ID	Unique integer IDs used to link each habitat image to its corresponding image.
Column B: List of Habitats	Names of the habitats and a radio button indicating the weight for each habitat.
Column C: Natural Mortality Rate	Importance weight of the average natural mortality rate with choices of: high mortality (e.g. 80% or higher), moderate mortality (e.g. 20-50%), low mortality (e.g. 0-20%), or no score (excludes from assessment).
Column D: Recruitment Pattern	Importance weight for the rate of recruitment or replacement of a species in a habitat with choices of: annually or more often, every 1-2 years, every 2+ years, or no score (excludes from assessment).
Column F: Connectivity	Importance weight for biotic habitats only representing the dispersal distance of propagules with choices of: low dispersal, medium dispersal,high dispersal, and no score (excludes from assessment).
Column G: Age of Maturity	Importance weight for biotic habitats only representing the habitat recovery time to a mature state with choices of: less important, equal importance, more important, and unrated.

**Worksheet (3)**	
Column A: Habitat ID	Unique integer IDs used to link each habitat image to its corresponding image.
Column B: Habitat	Names of the habitats that match names used in the previous worksheets.
Column C: Stressor ID	Stressor ID used in the previous worksheets linking the stressor to the habitat.
Column D: Stressor	Names of the stressor that match names used in the previous worksheets.
Column F: Change in Structure	Importance weight for the loss of ecosystem structure for the habitat (column A as a result of the stressor (column B) with choices of: low loss, medium loss, high loss, and no score (excludes from assessment).
Column G: Natural Disturbance Frequency	The frequency of perturbation in the habitat.
Column H: Overlap Time	The amount of time that the stressor and habitat overlap.

-

Worksheet Rating Legends

Contains information on the names of the categories in the drop-down menus used in the previous worksheets. The names of the categories (Column C) can be changed if desired. The spatial overlap threshold values (Column D) qualifies the effect of stressor on habitat based on its overlap. The proportion of the landscape that should overlap to be considered categories 1,2, or 3 can be changed by the user - that will automatically change the caption of Column C.

Worksheets Calc (Exp) and Calc (con)

These last two worksheets are used by the model to perform the calculations and should not be modified by the user.

3. Several outputs are created when the model is run. For each habitat the cumulative risk output map shows the cumulative risk of all stressors on that habitat. It is the sum of all risk scores from each stressor. The "ecorisk" output map is the cumulative risk scores for all habitats. Finally, the "recov" output is the recovery potential from human activity for all habitats. A low recovery potential, for example, define areas that are highly vulnerable to human activities.
